# Do submerged macrophyte species influence crustacean zooplankton functional group richness and their resource use efficiency in the low-light environment?

**DOI:** 10.3389/fpls.2023.1185947

**Published:** 2023-06-06

**Authors:** Li Wang, Xufa Ma, Jun Chen

**Affiliations:** ^1^College of Fisheries, Huazhong Agricultural University, Wuhan, China; ^2^Donghu Experimental Station of Lake Ecosystems, State Key Laboratory of Freshwater Ecology and Biotechnology, Institute of Hydrobiology, Chinese Academy of Sciences, Wuhan, China

**Keywords:** zooplankton grazing, submerged macrophytes, low-light stress, plant density, ecological function

## Abstract

During the high grazing of epiphytic zooplankton in submerged macrophyte beds, the changes in crustacean zooplankton functional groups are crucial for stabilizing a clear water state in shallow lakes. However, submerged macrophytes often experience low-light stress due to many ecological processes. It is unclear whether submerged macrophytes alter the zooplankton functional group and their resource use efficiency in the low-light environment. We conducted two mesocosm experiments involving the treatments of low-light and submerged macrophyte species (*Vallisneria natans* and *Potamogeton maackianus*). The results show that abiotic factors (e.g., light) were the most important variables in explaining the change in the zooplankton community. Specifically, zooplankton functional group (i.e., pelagic species, plant-associated species, and substrate scrapers) richness and zooplankton species diversity decreased with the decreasing light intensity, especially for low substrate scraper abundance. In addition, structural equation models showed that low-light stress reduced zooplankton resource use efficiency by reducing zooplankton functional group richness and species diversity. Compared to species diversity, zooplankton functional group richness had a greater influence on their resource use efficiency (Zp/Chl-*a*) in the low-light environment. Our results suggest that the low-light stress reduced zooplankton resource use efficiency by changing their functional group richness. Moreover, the abundance of substrate scrapers shaken from *V. natans* was higher than that from *P. maackianus*. Therefore, submerged macrophyte species influence crustacean zooplankton functional group richness and their resource use efficiency in the low-light environment. Selecting appropriate aquatic plant species to assure the high diversity of zooplankton should be considered when conducting lake restoration using submerged macrophytes.

## 1 Introduction

Submerged macrophytes are a key functional group in lake ecosystems (Jeppesen et al., 1997). However, many submerged macrophytes in lakes have declined or even disappeared in recent years in China and worldwide, and there are still many difficulties in recovering all submerged macrophytes in lakes due to the unclear recession mechanism (Qin et al., 2014; Zhang et al., 2017). One of the most important factors is the ubiquitous decrease in underwater light availability, limiting the growth of submerged macrophytes (Chen et al., 2016; Jin et al., 2020). The underwater light availability is also critical for determining freshwater biodiversity in submerged macrophyte-dominated lakes (Karlsson et al., 2009; Estlander et al., 2017; Yu et al., 2021). The lowest zooplankton species richness and total abundance were in open water, while the highest richness and abundance were within the *Chara tomentosa* stand (Kuczyńska-Kippen and Joniak, 2016). Substrate scrapers (e.g., *Alona guttata or Chydorus sphaericus*) usually have particularly high densities in ecosystems where macrophytes are extensively developed (Blindow, 1987). High-light supplementation not only enables the growth of *Vallisneria natans* but also increases the periphyton attached to the leaves (Yu et al., 2021). However, our understanding of the effect of submerged macrophytes on crustacean zooplankton functional groups in the low-light environment remains largely unknown.

Zooplankton play a key role in maintaining freshwater biodiversity and in grazing algae (Wang et al., 2022). Previous studies of submerged macrophytes on zooplankton have mainly examined the changes in species in open water *vs.* the littoral zone of shallow lakes (Kuczyńska-Kippen et al., 2009; Kuczyńska-Kippen and Joniak, 2016; Kuczyńska-Kippen, 2018a; Kuczyńska-Kippen and Malgorzata, 2018b). Many ecologists agree that complex community structures can be simplified by categorizing species into functional groups based on suites of correlated traits (Pennak, 1966; Lavorel et al., 1997). Depending on the different habitat and feeding preferences, crustacean zooplankton functional group composition (the types of functional groups) includes pelagic species, plant-associated filter feeders, and substrate scrapers (Pennak, 1966; Fryer, 1968; Downing, 1981). Abiotic factors are commonly demonstrated as the major determinant in structuring zooplankton diversity in freshwater ecosystems (Heino et al., 2017). Light influences the horizontal migratory behavior of plant-attached crustacean zooplankton in open water and the littoral zone in the water body with floating macrophytes (Estlander et al., 2017). Weak light also reduces the photosynthesis of submerged macrophytes to decrease the dissolved oxygen (DO) concentration and pH of water (Vilas et al., 2017), mediating trophic interactions between zooplankton and their habitat environment (Reeder, 2011; Zhao et al., 2013; Wang et al., 2018). Moreover, macrophytes with different life forms have different low-light adaptation strategies via altering photosynthetic and morphological adaptations (Chen et al., 2016; Yuan et al., 2016). The habitat heterogeneity created by macrophyte morphological structures could influence the attachment for substrate scrapers (Choi et al., 2014a; Choi et al., 2014b; Choi et al., 2015). Therefore, light may influence zooplankton functional group richness (the number of functional groups) and species diversity in the zone of submerged macrophytes.

Resource use efficiency is an ecological concept that quantifies ecological function by measuring the proportion of supplied resources and converted biomass from organisms (Ptacnik et al., 2008; Hodapp et al., 2019). Chlorophyll-*a* (Chl-*a*) is a proxy of the supplied resources. Zooplankton resource use efficiency (e.g., zooplankton grazing on phytoplankton) is calculated as the ratio of zooplankton biomass and Chl-*a* (Jeppesen et al., 2000). Recently, the relationships between biodiversity and ecological functions have been the focus. Positive biodiversity–ecological functional relationships have been reported in different types of aquatic ecosystems (Ye et al., 2013; Thompson et al., 2015; Yang et al., 2022). Littoral macrophyte zones are heterogeneous environments with high zooplankton species diversity (Masclaux et al., 2014) and may have high resource use efficiency (Wang et al., 2023). In shallow lakes, the high grazing of epiphytic zooplankton in submerged macrophyte beds is the key mechanism, explaining the positive role of plants in stabilizing a clear water state (Balayla and Moss, 2004). It is not clear whether zooplankton functional group richness or zooplankton species diversity is a better indicator of their resource use efficiency in the low-light environment.

Here, we conducted two controlled experiments to explore the responses of crustacean zooplankton to low-light stress in two submerged macrophyte systems (*V. natans* and *Potamogeton maackianus*). We tested the predictions that i) low-light stress reduces epiphytic species abundance and species diversity, which may weaken their resource use efficiency; ii) compared to species diversity, functional group richness is a better indicator of resource use efficiency in the low-light environment.

## 2 Methods

### 2.1 Materials

*V. natans* and *P. maackianus* are the dominant species in the lakes of the middle and lower reaches of the Yangtze River, in China (Su et al., 2019). *V. natans* belongs to the rosette growth form, with its long and narrow tape-shaped leaves allowing more light penetration and nutrient exchange (Chou et al., 2022). *P. maackianus*, as a canopy former, mainly elongates its shoot length toward the water surface to compensate for the low-light conditions (Chen et al., 2016). As *V. natans* has a bigger leaf per unit mass, it can encourage periphyton growth and large zooplankton communities of plant-associated filters (Hao et al., 2017).

*P. maackianus* (height of 31.72 ± 8.72 cm) and *V. natans* (height of 30.0 ± 3.1 cm) were cultured in outdoor aquaria (length, 50 cm; width, 50 cm; height, 80 cm) filled with 70 cm of mixed water (a mixture of 30% water from Lake Donghu including zooplankton and 70% tap water) at the Donghu Experimental Station of Lake Ecosystem (30°32′53.41″N, 114°21′15.63″E), Wuhan City, China.

At the beginning of the experiment, submerged macrophyte shoots/seedlings were carefully washed to remove the attachments before being planted in tanks. To keep macrophytes growing in the ideal culture conditions, we diluted the water from Donghu Lake with tap water to decrease the total phosphorus (TP) concentration to below 0.08 mg/L. The sediment was collected from the same location in the Tanglin area of Donghu Lake and intensively mixed before being divided evenly into boxes or cups.

### 2.2 Experimental design

#### 2.2.1 Low-light controlled experiment (L-Experiment)

**Figure 1:**
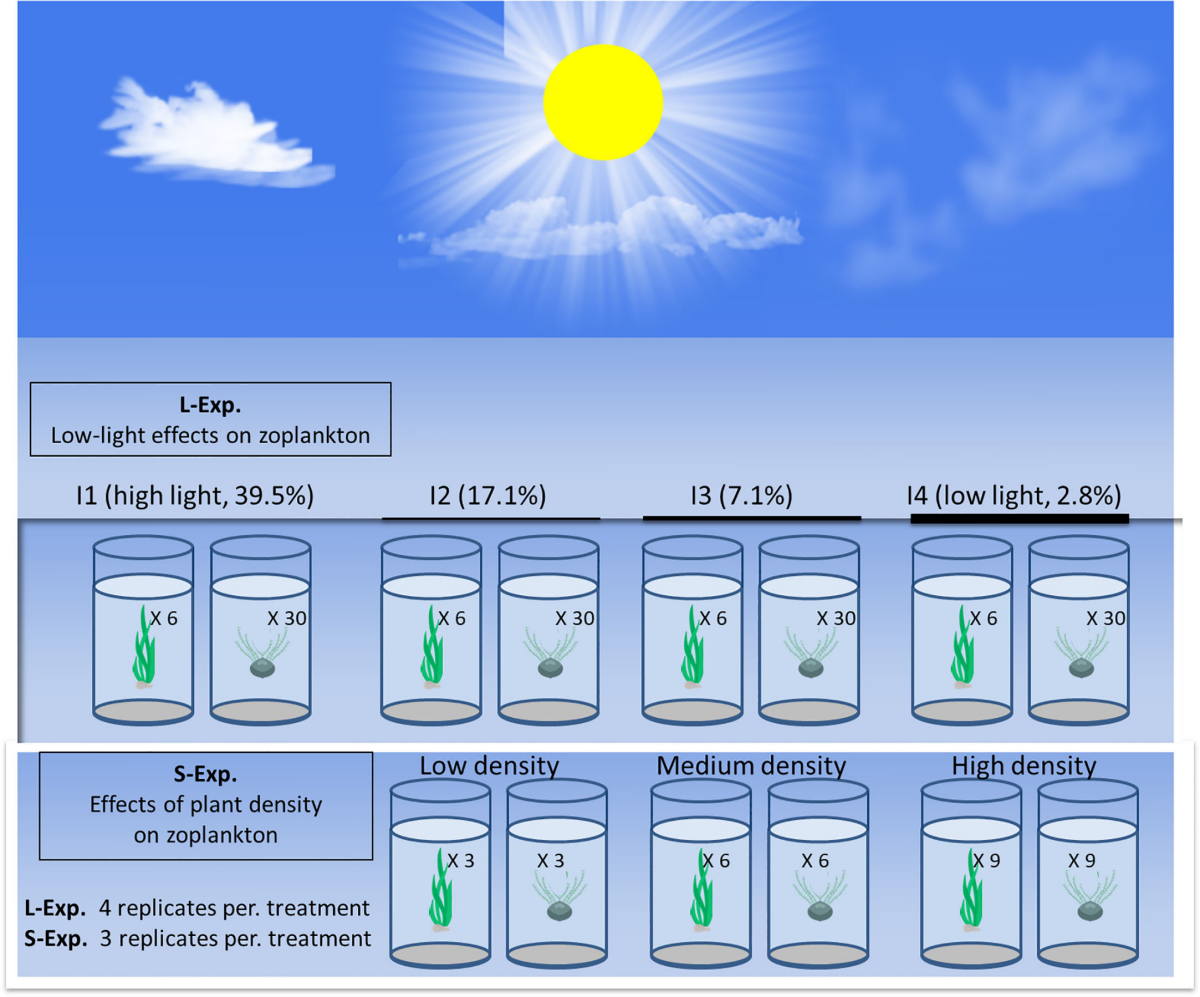
Experiment layout for the *Vallisneria natans* and *Potamogeton maackianus* cultivation experiments. Aquaria (50 × 50 × 80 cm) filled with 70 cm of mixed water (a mixture of 30% water from Lake Donghu (including zooplankton) and 70% tap water). Each cup contained one *P. maackianus*, and the boxes contained various *V. natans* individuals.

The L-Experiment was conducted from 28 May to 20 November 2015. The water from each aquarium was sampled once every 2 weeks. *V. natans* and *P. maackianus* were cultured in 32 aquaria. Each *V. natans* tank contained two plastic boxes (length, 40 cm; width, 25 cm; height, 12 cm) with three healthy seedlings of *V. natans* individuals and 10 cm sediment. Each *P. maackianus* tank contained 30 cups with one healthy shoot of *P. maackianus* individual in a plastic cup (diameter, 6.5 cm; height, 9.8 cm) and 9 cm sediment. Those aquaria were placed in a shelter covered by black nylon nets of various thicknesses, resulting in four light regimes [i.e., 39.5% (I1, high light), 17.1% (I2), 7.1% (I3), and 2.8% (I4, low light)], reflecting the natural light intensities in the aquaria (**Figure 1**). Each treatment included four replicates.

#### 2.2.2 Submerged macrophyte density controlled experiment (S-Experiment)

Low-light stress may directly or indirectly affect the zooplankton community by inhibiting the growth of freshwater macrophytes. To study the influence of submerged macrophyte density without low-light stress disturbing epiphytic zooplankton, the S-Experiment was conducted from 26 September to 25 November 2017. The water of each aquarium was sampled once every 10 days. *V. natans* and *P. maackianus* were cultured in 21 outdoor aquaria. Healthy *V. natans* ramets (31.72 ± 8.42 cm height) and *P. maackianus* shoots (30.72 ± 5.72 cm height) were planted evenly at three densities of three, six, and nine plants in one plastic box per aquarium [seven treatments: C0 (no plants), V1 (three *V. natans*), V2 (six *V. natans*), V3 (nine *V. natans*), P1 (three *P. maackianus*), P2 (six *P. maackianus*), and P3 (nine *P. maackianus*)] (**Figure 1**). Each treatment included three replicates.

### 2.3 Measurement of environmental variables, submerged macrophytes, and crustacean zooplankton communities

#### 2.3.1 Environmental variables

A Plexiglas tube (length, 1 m; diameter, 70 mm) was used to drain the water out of the aquaria (Nicolle et al., 2012). From the top to the bottom of each aquarium, 5 L of mixed water was obtained using a Plexiglas tube. A 1-L water sample was taken to the laboratory for total nitrogen (TN), NO_3_, NH4, TP, PO_4_, and chlorophyll-*a* (Chl-*a*) analyses according to the methods described by Clesceri et al. (1998). Water temperature (T), pH, and DO were measured in the overlying water using a multifunctional YSI meter (Yellow Springs Instruments, OH, USA) in the morning. Photosynthetic active radiation (PAR) near the water surface (depth, 0 m) within the water column was measured at every sampling time (once for three to four replicates) by a Li-1400 data logger (Li-Cor, Lincoln, NE, USA) from 10:00 a.m. to 12:00 a.m.

#### 2.3.2 Submerged macrophytes

The leaves of *V. natans* and the stems and leaves of *P. maackianus* were harvested, gently washed, dried with tissue paper, and weighed to determine the dry weight at each sampling site. One seedling was harvested three times on the 30th, 60th, and 90th days of the L-Experiment. At the beginning of the S-Experiment, the submerged macrophyte leaf area per shoot/seedling was estimated using ImageJ software; at the end of the S-Experiment, all plants of each aquarium were harvested and carefully washed with tap water for dry weight.

The macrophytes’ relative change rate was calculated by the ratio of the difference in macrophyte wet weight and days. It could provide information on the ecological adaptation of submersed macrophytes in response to a varying environment.

#### 2.3.3 Crustacean zooplankton

The remaining 4 L of mixed water was filtered through a 64-μm plankton net and preserved with formaldehyde solution for later crustacean zooplankton analyses in both the L-Experiment and the S-Experiment. All individuals were identified and counted to species level for each sample under ×40 magnification using a light microscope (Olympus BX21, Tokyo, Japan) according to the methods by Shen et al. (1979) and Chiang and Du (1979). The biomass of each species was calculated based on zooplankton volume (Huang et al., 1999).

Moreover, as epiphytic zooplankton need substrate surfaces for attachment (Choi et al., 2014a), their abundance is often underestimated in the traditional collected method (Timms and Moss, 1984; Nurminen et al., 2001). Therefore, at the end of the S-Experiment, the collected *V. natans* and *P. maackianus* were shaken vigorously 30, 60, and 90 times. All the fallen zooplankton were collected after every 30 shakes in a plastic bag containing water, filtered through a 64-µm plankton mesh, and preserved with formaldehyde solution for later enumeration at ×40 magnification.

We first categorized 16 zooplankton species into four functional traits based on data from specialized literature (**Table S1**) to calculate functional diversity. The selected functional traits described the morphology, physiology, and ecology of zooplankton species.

Functional groups are used to describe patterns of community organization. They are defined either by suites of correlated traits or by species groupings. Morphological and physiological characteristics in zooplankton are correlated with an adaptive response to environmental conditions. Based on the habitat and feeding preferences of **Table S1**, the zooplankton functional group and species added were as follows: pelagic species *Diaphanosoma brachyurum*, *Sinocalanus dorrii*, *Daphnia galeata*, *Leptodora kindti*, *Mesocyclops leuckarti*, and *Thermocyclops taihokuensis*; plant-associated species *Camptocercus rectirostris*, *Scapholeberis mucronata*, *Eucylops serrulatus*, *Sida crystallina*, and *Simocephalus vetulus*; substrate scrapers *A. guttata*, *Pleuroxus laevis*, *C. sphaericus*, and *Alonella excisa* in this study.

### 2.4 Statistical analysis

#### 2.4.1 Drivers of crustacean zooplankton species diversity and composition

The species alpha diversity indices including Shannon–Wiener, Simpson, and Pielou evenness (J) were calculated by zooplankton abundance data using the “vegan” package (Oksanen et al., 2013) in R software (R core team, 219). Functional diversity indices, including functional richness (Fric), functional dispersion (FDis), and RaoQ, were calculated using crustacean zooplankton abundance data and functional traits (see **Table S1**) by the “FD” package (Laliberté et al., 2014) in R software.

To investigate the drivers of crustacean zooplankton species diversity and composition in the S-Experiment, we conducted variation partitioning analysis (VPA) to reveal the relative importance of three explanatory datasets [i.e., abiotic factors (light intensity, pH, DO, temperature, TDS, and conductivity), nutrients (TN, NO_3_, NH4, TP, and PO_4_), and food (plant dry weight and Chl-*a*)] to zooplankton species diversity and composition using the “varpart” function (Lai et al., 2021). Adjusted-r^2^ values for all analyses were reported as unbiased estimates of the explained variation. We also tested for the significance of pure fractions of each explanatory variable dataset by means of 999 permutations at a significance level of α = 0.05 using the “anova.cca” function in the “vegan” package (Oksanen et al., 2013). Abiotic factors (e.g., light intensity) were the most important variables in explaining the zooplankton species’ alpha diversity and composition, but not functional diversity.

#### 2.4.2 Crustacean zooplankton species diversity and composition analyses under various light regimes

As the density of macrophytes in each aquarium increased during the experimental periods, these observations were not independent in the L-Experiment and S-Experiment. Then, the time-series data were statistically tested for the effect of treatments, time, and their interaction by repeated-measures ANOVA (rmANOVA) in SPSS (IBM SPSS Statistic 20), including values for environment variables, zooplankton species alpha diversity, and functional groups. Before analysis, data were log10(x + 1) (if a value of zero existed) transformed to meet assumptions of normality or homogeneity of variance. If the assumption of sphere city was violated in the variance–covariance matrices of rmANOVA analyses, the degrees of freedom (df) were Huynh–Feldt corrected, resulting in an adjustment of the significance of the F ratio (Huynh and Feldt, 1976). In the event of significant interaction terms, a *post-hoc* pairwise comparison was performed (Holm–Sidak method) to determine where the differences occurred, using light treatments as a categorical factor and light intensity as a quantitative factor.

#### 2.4.3 Correlation of crustacean zooplankton resource use efficiency with light

Principal component analysis (PCA) was used to resolve the co-linearity and generate the axis one score of species alpha diversity (PC1_diversity_) and the axis one score of functional group abundance (PC1_functional_) based on the species alpha diversity indices and functional group abundance, respectively (see Section 1 in the **Supplementary Material** for details).

Structural equation models (SEMs; function “sem” in R package “lavaan”) were used to explore how zooplankton functional groups and species alpha diversity influenced their resource use efficiency in the two kinds of macrophyte groups. SEM analyses were constructed based on four variables in the L-Experiment and S-Experiment: light intensity, PC1_diversity_, PC1_functional_, and the ratio of zooplankton biomass and Chl-*a*. The root-mean-square error of approximation (RMSEA) and the standardized root-mean-square residual (SRMR) with values less than 0.05 were used to determine the goodness of fit of the model (Schermelleh-Engel et al., 2003).

## 3 Results

### 3.1 Environmental variables

The Chl-*a* concentration remained relatively low and showed no significant difference (*p* > 0.05) between treatments in the L-Experiment (**Tables 1**, **S2**). DO (*p* < 0.001), pH (*p* < 0.001), and TN (*p* < 0.05) showed significant differences among the light treatments and exhibited the same tendencies over the sampling time. Post-hoc multiple comparisons showed significant differences in light intensity between any two treatments. In contrast, DO, pH, TN, TP, and Chl-*a* concentrations showed no significant difference (*p* > 0.05) among the treatments in the S-Experiment (**Tables 1**, **S2**).

**Table 1:** Summary of repeated-measures analysis of variance (rmANOVA) results for the effect of different light (Light) and plant species (Plant) scenarios on environmental variables during the experiments (Time).

	Light intensity			pH			DO			Chl-*a*			TN			TP		
	DF	F	p	DF	F	p	DF	F	p	DF	F	p	DF	F	p	DF	F	p
Plant	1	7.022	**<0.05**	1	6.582	**<0.05**	1	4.065	0.057	1	1.761	0.199	1	2.086	0.163	1	7.288	**<0.05**
Light	3	425.564	**<0.001**	3	36.151	**<0.001**	3	220.06	**<0.001**	3	1.56	0.229	3	3.236	**<0.05**	3	2.828	0.063
Plant × Light	3	0.474	0.704	3	4.06	**<0.05**	3	25.388	**<0.001**	3	1.514	0.24	3	0.869	0.473	3	1.172	0.344
Time	2.794	37.514	**<0.001**	1	142.424	**<0.001**	4.932	170.847	**<0.001**	1.043	1.431	0.246	2.089	3.471	**<0.05**	2.797	10.568	**<0.001**
Time × Plant	2.794	2.139	0.109	1	7.97	**<0.05**	4.932	9.605	**<0.001**	1.043	1.625	0.217	2.089	0.809	0.457	2.797	3.004	**<0.05**
Time × Light	8.382	8.325	**<0.001**	3	32.875	**<0.001**	14.797	9.485	**<0.001**	3.13	1.536	0.215	6.268	3.44	**<0.01**	8.391	2.391	**<0.01**
Time × Plant × Light	8.382	2.163	**<0.05**	3	3.622	**<0.05**	14.797	1.51	0.116	3.13	1.583	0.221	6.268	1.287	0.26	8.391	1.436	0.198

Values indicate probability levels; values in bold are below the significance level (0.05).

DO, dissolved oxygen; Chl-a, chlorophyll-a; TN, total nitrogen; TP, total phosphorus.

### 3.2 Submerged macrophytes

During the L-Experiment period, the *P. maackianus* relative change rates were positive in the high-light groups (I1–I2) and negative in the low-light groups (I3–I4). In contrast, the *V. natans* relative change rates were still positive even in the low-light groups (I3–I4) (**Figure S1a**). During the S-Experiment period, the relative change rate of two macrophytes showed no density gradient distribution (**Figure S1b**).

The light treatments significantly affected the total dry biomass of *P. maackianus* and *V. natans* in the L-Experiment (**Table S2**). The total biomass increased with the light intensity available; the largest values were obtained in the I1 and I2 groups, and the lowest values (V, 0.62 ± 0.09 g; P, 0.64 ± 0.09 g) were obtained in the I4 group (**Table S2**). At the beginning of the S-Experiment, the macrophyte leaf area of one *V. natans* seedling was much higher than that of one *P. maackianus* shoot (**Figure S2**). At the end of the S-Experiment, the total dry biomass of *P. maackianus* and *V. natans* still showed a density gradient distribution (i.e., low-, medium-, and high-density levels; **Table S2**).

### 3.3 Crustacean zooplankton functional groups

**Figure 2:**
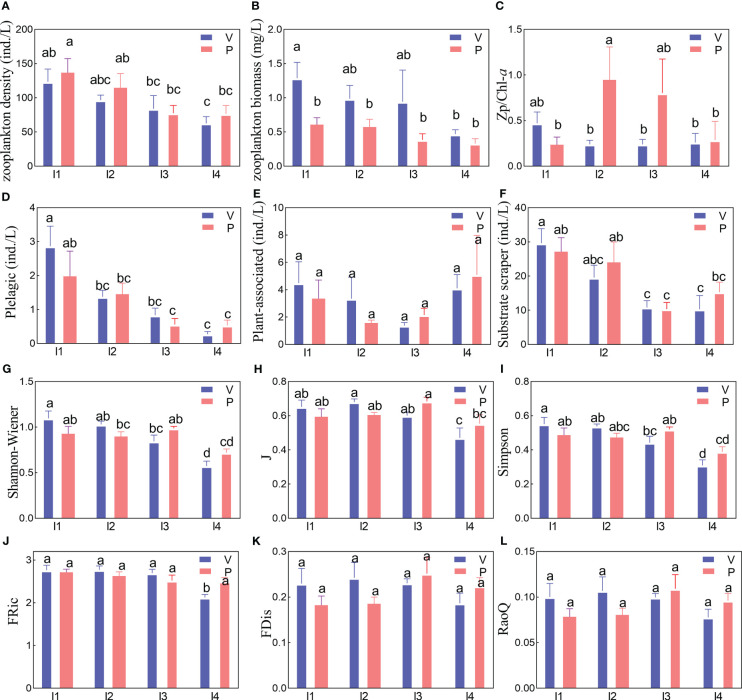
Variations in crustacean zooplankton density **(A)**, biomass **(B)**, Zp/Chl-*a* **(C)**, functional groups density **(D–F)**, species alpha diversity **(G–I)** and functional diversity **(J–L)** in the four light treatments (I1–L4). Bars represent an average of replicates ( ± SE). I1 (high light), I2, I3, and I4 (low light) represent 39.5%, 17.1%, 7.1%, and 2.8% natural light in the aquaria, respectively. V and P represent *Vallisneria natans* and *Potamogeton maackianus*, respectively. Functional richness, Fric; functional dispersion, FDis; Pielou evenness, J; the ratio of zooplankton biomass and Chl-*a*, Zp/Chl-*a*.

In the L-Experiment, light intensity significantly affected total zooplankton density and zooplankton functional group abundances (*p* < 0.05), but not for total zooplankton biomass and Zp/Chl-*a* in seston (*p* > 0.05) (**Table 2** and **Figures 2A–F**). Zooplankton density in the *V. natans* group was lower than that in the *P. maackianus* group, but the change in zooplankton biomass had an opposite trend (**Figures 2A, B**). Pelagic species and substrate scrapers exhibited different variation tendencies over time (**Table 2**). In contrast, zooplankton functional groups in seston showed no significant difference (*p* > 0.05) among the treatments in the S-Experiment (**Table S3**). The highest zooplankton density (141.35 ± 15.69 ind./L) appeared in the medium-density level of *V. natans*, and zooplankton biomass was the highest (0.87 ± 0.16 mg/L) in the high-density level of *P. maackianus* (**Table S3**).

**Table 2:** Summary of repeated-measures analysis of variance (rmANOVA) results for the effect of different light (Light) and plant species (Plant) scenarios on total crustacean zooplankton density and biomass and the density of three functional groups during the experiments (Time).

	Total density			Total biomass			Pelagic species			Plant-associated species			Substrate scrapers		
	DF	F	p	DF	F	p	DF	F	p	DF	F	p	DF	F	p
Plant	1	2.665	0.118	1	5.897	**0.024**	0.486	0.072	0.79	1	0.005	0.945	1	1.635	0.215
Light	3	8.783	**0.001**	3	2.035	0.14	3	11.685	**<0.001**	3	1.334	0.29	3	11.22	**<0.001**
Plant × Light	3	0.666	0.583	3	0.38	0.796	3	0.376	0.771	3	0.438	0.728	3	0.415	0.744
Time	4.74	8.195	**<0.001**	3.245	3.202	**0.025**	3.649	3.31	**0.018**	3.493	0.829	0.498	4.383	5.276	**<0.001**
Time × Plant	4.74	1.305	0.27	3.245	3.524	**0.017**	3.649	1.535	0.205	3.493	0.836	0.493	4.383	1.33	0.263
Time × Light	14.219	1.238	0.26	9.734	0.611	0.796	10.947	1.284	0.25	10.48	1.319	0.234	13.148	1.429	0.161
Time × Plant × Light	14.219	0.895	0.567	9.734	0.679	0.737	10.947	0.735	0.702	10.48	1.345	0.221	13.148	0.798	0.662

Values indicate probability levels; values in bold are below the significance level (0.05).

In the S-Experiment, whether in seston or macrophytes, substrate scraper was the dominant zooplankton functional group (**Table S4** and **Figure S3a**). The density of substrate scraper in the *V. natans* group was higher than in the *P. maackianus* group (**Figure S3a**). *S. crystallina* attached tightly to *V. natans* and appeared only when the collected macrophytes were shaken more than 30 times vigorously (**Figure S3b**).

### 3.4 Crustacean zooplankton species diversity and composition

In the L-Experiment, zooplankton species alpha diversity showed significant differences (*p* < 0.05) among the light treatments and exhibited different tendencies over the sampling time, but not for functional diversity (**Figures 2G–L** and **Table 3**). The species alpha diversity increased with the light intensity increase. The highest species alpha diversity was obtained in the I1 group (**Figures 2G–I** and **Table 3**). In contrast, in the S-Experiment, zooplankton species alpha diversity showed no significant differences (*p* > 0.05) among the plant density treatments (**Table S3**).

**Table 3:** Summary of repeated-measures analysis of variance (rmANOVA) results for the effect of different light (Light) and plant species (Plant) scenarios on crustacean zooplankton species diversity during the experiments (Time).

	J			Shannon–Wiener			Simpson			Fric			FDis			RaoQ		
	DF	F	p	DF	F	p	DF	F	p	DF	F	p	DF	F	p	DF	F	p
Plant	1	0.139	0.713	1	0.281	0.602	1	0.04	0.843	1	0.429	0.519	1	0.628	0.437	1	0.589	0.451
Light	3	4.168	**0.018**	3	8.939	**0.001**	3	7.355	**0.001**	3	7.703	**0.001**	3	1.021	0.403	3	1.035	0.398
Plant × Light	3	3.23	**0.043**	3	3.701	**0.028**	3	3.068	**0.05**	3	4.037	**0.021**	3	1.578	0.225	3	1.753	0.187
Time	11	2.328	**0.01**	6.108	3.465	**0.003**	6.118	3.054	**0.008**	11	1.09	0.37	6.229	3.719	**0.002**	6.253	3.952	**0.001**
Time × Plant	11	1.629	0.092	6.108	2.224	**0.044**	6.118	1.95	0.076	6.65	0.789	0.651	6.229	1.553	0.163	6.253	1.53	0.17
Time × Light	33	1.762	**0.009**	18.325	1.908	**0.02**	18.353	2.007	**0.013**	19.951	2.015	**0.002**	18.686	2.28	**0.004**	18.759	2.225	**0.005**
Time × Plant × Light	33	1.748	**0.01**	18.325	1.312	0.19	18.353	1.431	0.126	33	0.703	0.887	18.686	1.259	0.223	18.759	1.262	0.221

Values indicate probability levels; values in bold are below the significance level (0.05).

Fric, functional richness; FDis, functional dispersion; J, Pielou evenness.

**Figure 3:**
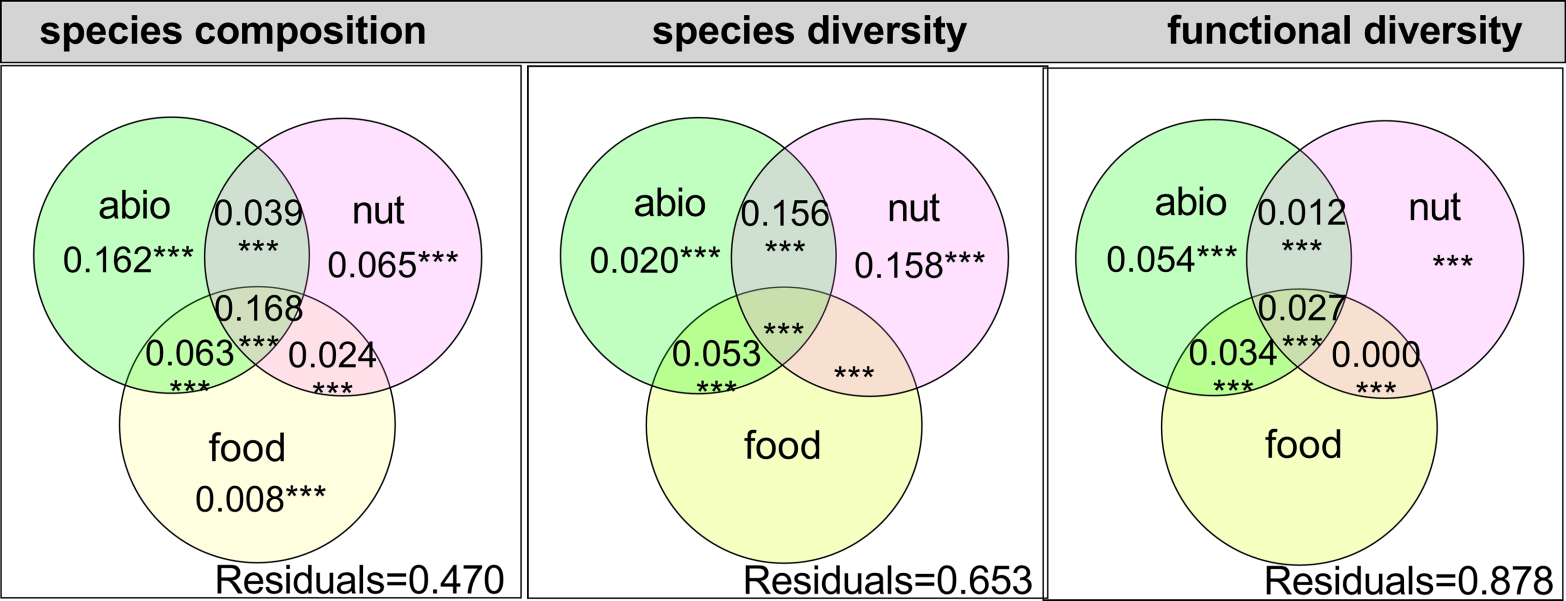
Three Venn diagrams illustrating the results of variation partitioning for crustacean zooplankton species composition, species alpha diversity, and functional diversity. Values in the circles indicate the variation in the zooplankton functional groups by abiotic factors (abio), nutrients (nut), food, and shared components. Residuals are shown in the lower right corner. All fractions (***p < 0.001) are based on adjusted R^2^ values shown as percentages of the total variation.

Overall, the results of VPA showed that abiotic factors, nutrient variables, and food explain the species composition of zooplankton (Fraction = 53.0%, **Figure 3**) and species alpha diversity (Fraction = 34.7%, **Figure 3**), but not for functional diversity (Fraction = 12.2%, **Figure 3**). In addition, abiotic factors and nutrient variables were the two most vital factors in explaining the zooplankton species alpha diversity and composition (**Figure 3**).

### 3.5 Crustacean zooplankton resource use efficiency

**Figure 4:**
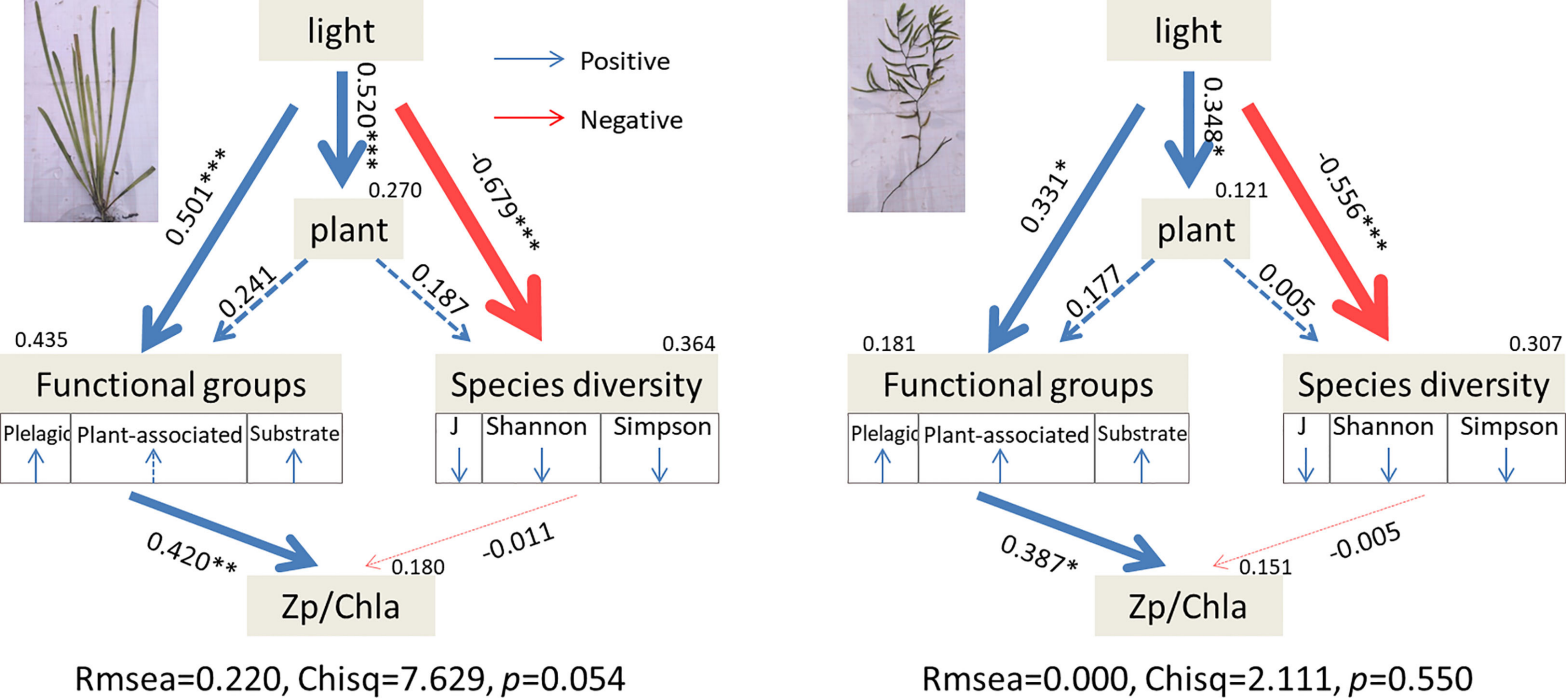
Path diagrams of the structural equation model (SEM) based on the interactions between light intensity, plant dry weight, crustacean zooplankton functional groups, and crustacean zooplankton species diversity. Boxes represent observed variables; arrows point from independent to dependent variables; numbers on the arrows correspond to the standardized path coefficients. Significant and non-significant path coefficients are indicated by full and dotted lines, respectively. The red and black arrows indicate positive and negative flows of causality (*p* < 0.05), respectively. Double-layer rectangles represent the first component from the PCA conducted for functional groups and species diversity. Functional groups include pelagic species, plant-associated species, and substrate scrapers as indicated by PCA; species diversity includes Shannon, Simpson, and Pielou. The red “↑” and blue “↓” symbols indicate positive and negative relationships between the variables and the first component from the PCA, respectively. The asterisks indicate very high significance, *p* < 0.001 (***), high significance, *p* < 0.01 (**), and significance, 0.01 < *p* < 0.05 (*). PCA, principal component analysis.

The results of SEM showed that light intensity had a direct and indirect influence on zooplankton functional group richness. Moreover, the indirect effect (V: standardized path coefficient = 0.520, 0.241; P: standardized path coefficient = 0.348, 0.177) was greater than the direct effect (V: standardized path coefficient = 0.501; P: standardized path coefficient = 0.331). Then, zooplankton functional group richness had a greater influence on the ratio of zooplankton biomass and Chl-*a* than species alpha diversity in both *V. natans* (standardized path coefficient = 0.420, *p* < 0.01) and *P. maackianus* (standardized path coefficient = 0.387, *p* < 0.05). Moreover, light had a greater influence on *V. natans* (standardized path coefficient = 0.520, *p* < 0.001) than on *P. maackianus* (standardized path coefficient = 0.348, *p* < 0.05) (**Figure 4**).

## 4 Discussion

Our results support the predictions of our hypothesis (i), i.e., that low-light intensity decreases epiphytic species abundance and species diversity, which may weaken their resource use efficiency. It may be that, on the one hand, light intensity positively affects the density of substrate scrapers attached to floating leaves but negatively impacts the density in water to save considerable energy (Fairchild, 1981; Nurminen and Horppila, 2006; Estlander et al., 2017). However, on the other hand, low-light stress indirectly influences littoral zooplankton by changing the growth of submerged macrophytes. Zooplankton prefers to live in environments with higher DO concentration and pH (Choi et al., 2014a). Low-light stress decreased DO and pH to suppress zooplankton abundance in the L-Experiment. It may be that low-light stress weakens macrophyte photosynthesis (Chen et al., 2016). In contrast, among the treatments of the S-Experiment, DO, pH, TN, TP, and Chl-*a* concentration as well as zooplankton abundance and species diversity in seston showed no significant difference. However, substrate scrapers shaken from the macrophytes were significantly larger than the other two groups. Though the plant density treatment was not successful in this study, the results indicated that macrophytes could change the variation of zooplankton functional group richness. Therefore, the indirect effect of light may play a bigger role in zooplankton functional group richness than the direct effect of light, which was also supported by the SEM results of two macrophyte groups.

Light availability is important to zooplankton resource use efficiency (Håll, 2013). Our study has indicated that low-light stress could reduce zooplankton resource use efficiency by changing their community. The trophic state is also crucial for the relationship between light and zooplankton resource use efficiency (Joniak et al., 2003; Kuczyńska-Kippen and Joniak, 2016). In mesotrophic ponds like in this study (TP, 0.02–0.04 mg/L; TN, 0.33–0.77 mg/L), light availability is relatively strongly related to zooplankton diversity (Kuczyńska-Kippen and Joniak, 2016), while in hypereutrophic ponds, a seemingly insignificant role of light is in the formation of high primary production in the subsurface layer (Joniak et al., 2003), which may be a signal of reduced control by zooplankton.

In the present study, water Chl-*a* was maintained at a relatively low concentration in all the treatments. This finding can probably be explained by a combination of those mechanisms. The first mechanism is the competition for light and water nutrients between macrophytes and phytoplankton because the growth of phytoplankton is mainly controlled by light and nutrient availability in the light limitation of nutrient-poor lake ecosystems (Karlsson et al., 2009). The second mechanism is low levels of turbulence in the mesocosms, which may also lead to lower populations of negatively buoyant phytoplankton. Lastly, allelopathy from *V. natans* may also inhibit the growth of phytoplankton (Jiang et al., 2019). Moreover, there were no fish in the experimental tanks. High grazing pressure from zooplankton might be one of the main reasons for the relatively low phytoplankton, with a high zooplankton:phytoplankton biomass ratio (Zp/Chl-*a*, 0.23–0.94).

Interestingly, zooplankton functional group richness performs better than species diversity in indicating resource use efficiency in two macrophyte groups, corroborating our hypothesis (ii). In contrast, many studies have shown that the positive biodiversity–ecological function relationships are more pronounced for measures of functional diversity and phylogenetic diversity than taxonomic diversity (Thompson et al., 2015). This inconsistency may be attributed to three reasons. First, the functional difference appeared to be well captured by our traits describing feeding type in this study. Different zooplankton subgroups employ different types of food resources; thus, grazing efficiency is high. Second, the traditional method collects a greater abundance of organisms within the water column; thus, substrate scrapers are underestimated as they cling to macrophytes tightly (Dodson et al., 2010), and the diversity of organisms is lower. Third, sampling is conducted in the different waterbodies under multiple disturbance events.

Both empirical and field studies have suggested that periphyton may compete with macrophytes as they absorb nutrients and light (Hill and Dimick, 2010), and their effect on macrophytes may be greater than that of phytoplankton (Sand-Jensen and Søndergaard 1981). Therefore, periphyton grazing is as important as suspension feeding (Balayla and Moss, 2004; in this study). Our study showed that there were no differences in the zooplankton community in the seston of two macrophyte species (**Table S3**). In contrast, for the zooplankton shaken from macrophytes, *V. natans* could support more periphyton grazers than *P. maackianus* (**Table S4**; V, 15.44 ind./tank; P, 6.15 ind./tank). It may be correlated with *V. natans* having a bigger leaf per seedling (**Figure S2**). Hao et al. (2017) suggested that *V. natans* have a bigger leaf, which can encourage periphyton growth and large zooplankton communities of plant-associated filters. Moreover, it was found that substrate scrapers attached more tightly to *V. natans* than to *P. maackianus* (**Figure S3b**). In the S-Experiment, we did not collect periphyton on the surface of the submerged macrophyte. However, according to the result of Zhang et al. (2019), the Chl-*a* content of epiphytic algae on the surfaces of *P. maackianus* is significantly higher than that on the surfaces of *V. natans*. Possibly, *V. natans* supports more periphyton grazers to enhance their grazing on the epiphytic algae. Species-specific preferences for different types of macrophytes indicate the high ecological value of macrophyte cover in ponds and a potential direction for the management of small water bodies toward maintaining a great variety of aquatic plants.

In aquatic ecosystems, there are many other biological processes influencing the shading of macrophytes. Benthic fish facilitate nutrient release from the sediment to promote the growth of phytoplankton and periphyton, reducing submerged macrophytes due to the shading effect (Chen et al., 2020a; Chen et al., 2020b). Ren et al. (2021) reported that snails consume periphyton and, thus, reduce their shading on the macrophytes. Therefore, the field lakes are complex, open, not simple, closed microcosm systems, making them hard to manage. We should consider the effects of low-light stress on zooplankton by combining various factors in the future. In addition, although we admitted that the chief concern of this study was that periphyton composition was not used for the resource use efficiency estimates, we wish to remind the readers of the limitations of applying the findings. Rotifers were not included, which may be insufficient to represent zooplankton. The amount of zooplankton in the plant density treatment were also not significantly different. We were still able to show how low-light stress affected the zooplankton functional group richness and the correlation between zooplankton functional group richness and their resource use efficiency. It could provide a guide for lake restoration using submerged macrophytes.

## Conclusions

Our results suggest that submerged macrophyte species influence crustacean zooplankton functional group richness and their resource use efficiency in the low-light environment. Specifically, low-light stress decreased zooplankton resource use efficiency mainly through suppressing plant-attached species. Hence, from a future perspective, pelagic species are likely to benefit more from low-light environments, whereas plant-attached species will likely suffer from even greater pressure in the future, imposed by the predicted decreased underwater light availability. Moreover, benthic fish promote nutrient release from the sediment to facilitate the shade effect of phytoplankton and periphyton. The potential effects of low-light stress on zooplankton resource use efficiency might further increase phytoplankton blooms through changing zooplankton functional group richness. Therefore, improving underwater light climate (such as increasing artificial light) and selecting proper aquatic plant species with larger specific leaf areas (such as *V. natans*) to assure high epiphytic zooplankton should be considered when conducting lake restoration using submerged macrophytes.

## Data availability statement

The raw data supporting the conclusions of this article will be made available by the authors, without undue reservation.

## Author contributions

LW originally formulated the research idea, performed data analyses, and wrote the manuscript. XM and JC commented on and edited the drafts of the manuscript. All authors contributed to the article and approved the submitted version.
