# Supplementary material for: Do submerged macrophyte species influence crustacean zooplankton functional group richness and their resource use efficiency in the low-light environment?
Source: Front Plant Sci. 2023 Jun 6;14:1185947. doi: 10.3389/fpls.2023.1185947 (PMC10280013; doi:10.3389/fpls.2023.1185947)
Supplement: Supplementary file 1 [file DataSheet_1.docx]

## **Supplementary Material**

**Do submerged macrophyte species influence crustacean zooplankton functional group richness and their resource use efficiency in the low-light environment?**

*Frontiers in Plant Science*

Li Wang^1,2^, Xufa Ma^1*^, Jun Chen^2*^

^1^ College of Fisheries, Huazhong Agricultural University, Wuhan 430070, China.

^2^ Donghu Experimental Station of Lake Ecosystems, State Key Laboratory of Freshwater Ecology and Biotechnology, Institute of Hydrobiology, Chinese Academy of Sciences, Wuhan, 430072, PR China.

*Corresponding author: Xufa Ma ([xufama@mail.hzau.edu.cn](mailto:xufama@mail.hzau.edu.cn)) and Jun Chen ([chenjun@ihb.ac.cn](mailto:chenjun@ihb.ac.cn))

**Section 1**

**The calculated method of axis one score of species** **alpha diversity indices and functional groups**

Both species alpha diversity indices (i.e., Shannon-Wiener, Simpson, and Pielou evenness) and functional groups (plelagic species, plant-associated species and substrate scrapers) were ordered using a PCA before conducting SEM. The linear correlation analysis between the species alpha diversity, functional groups and the axis one score was also tested. Within each group, the first component (PC1) explained over 90% of the total variance, then introduced as new variables into the subsequent SEM analysis and called the derived species alpha diversity, the derived functional groups. The method refers to Chen et al. (2016).

**Table S1** A list of crustacean zooplankton species surveyed in this study and their respective species traits.

| Species | Habitat | Body size | Trophic group | Feeding type |
| --- | --- | --- | --- | --- |
| *Diaphanosoma brachyurum* | Littoral | Medium | Herbivore | S-Filtration |
| *Sinocalanus dorrii* | Pelagic | Large | Herbivore | Stationary suspension |
| *Daphnia galeata* | Pelagic | Large | Herbivore | D-Filtration |
| *Leptodora Kindti* | Pelagic | Large | Carnivore | Raptorial |
| *Mesocyclops leuckarti* | Pelagic | Medium | Omnivore-Carnivore | Raptorial |
| *Thermocyclops taihokuensis* | Pelagic | Medium | Omnivore | Raptorial |
| *Alona guttata* | Littoral | Small | Herbivore | Substratum scrapers |
| *Pleuroxus laevis* | Littoral | Small | Herbivore | Substratum scrapers |
| *Camptocercus rectirostris* | Littoral | Medium | Herbivore | Substratum scrapers |
| *Scapholeberis mucronata* | Littoral | Medium | Herbivore | S-Filtration |
| *Eucylops serrulatus* | Littoral | Medium | Omnivore-Herbivore | Raptorial |
| *Sida crystallina* | Littoral | Large | Herbivore | S-Filtration |
| *Simocephalus vetulus* | Littoral | Large | Herbivore | S-Filtration |
| *Chydorus sphaericus* | Littoral | Small | Herbivore | Substratum scrapers |
| *Alonella excisa* | Littoral | Small | Herbivore | Substratum scrapers |
| *Ilyocryptus sordidus* | Benthic | Small | Herbivore | Detritivore-Herbivore |

Note: The first was habitat selection, classified into pelagic (open areas, preferably in the water column), littoral (associated to aquatic vegetation) and benthic (sediment substrates); the second trait was body size, classified into small, medium, and large; the third was trophic group, classified into herbivores, carnivore and omnivores (the organisms feed on algae, rotifers and cladocerans). The fourth was feeding type, cladocera had been divided into four classes: Daphnia-type filtraction (D), Bosmina-type (B), Sida-type (S), Chydoridae-type (C) and Ilyocriptidae-type (I), based on how they obtained food using their behavior and the structure of thoracic limbs (Barnett et al. 2007); copepods were characterized in stationary suspension (the search for food was passive and organisms swim less) and raptorial (predators that captured their prey). Information on species traits was obtained from previous studies (Chiang & Du, 1979; Shen et al., 1979; Barnett et al. 2007; Rizo et al., 2017; Braghin et al., 2021).

**Table S2** Variations in environment variables and submerged macrophytes dry weight.

|  |  | Groups | PAR  ( μmol m^-2^ s^-1^) | pH | TN  (mg/L) | TP  (mg/L) | Chl-*a*  (μg/L) | DO  (mg/L) | Temperature  (℃) | Plant dry weight (g) |
| --- | --- | --- | --- | --- | --- | --- | --- | --- | --- | --- |
| L-Experiment | V | I1 | 341.68±3.46^a^ | 9.50±0.04^a^ | 0.50±0.02^ab^ | 0.02±0.00^b^ | 2.15±0.34^a^ | 9.94±0.15^b^ | 26.00±0.08^a^ | 7.35±0.78^a^ |
|  |  | I 2 | 144.48±6.78^b^ | 9.28±0.02^b^ | 0.41±0.02^ab^ | 0.02±0.00^b^ | 2.35±0.38^a^ | 9.43±0.13^c^ | 25.77±0.13^b^ | 9.03±0.68^a^ |
|  |  | I 3 | 63.26±3.03^c^ | 8.91±0.04^d^ | 0.36±0.01^b^ | 0.02±0.00^b^ | 2.03±0.29^a^ | 8.56±0.06^d^ | 24.64±0.05^d^ | 4.21±0.31^b^ |
|  |  | I 4 | 20.93±0.72^d^ | 8.93±0.09^d^ | 0.47±0.01^ab^ | 0.02±0.00^b^ | 2.40±1.09^a^ | 7.72±0.04^e^ | 24.35±0.04^e^ | 0.62±0.09^c^ |
|  | P | I 1 | 348.71±18.60^a^ | 9.54±0.04^a^ | 0.77±0.31^a^ | 0.04±0.01^a^ | 18.06±14.86^a^ | 10.57±0.20^a^ | 25.77±0.13^b^ | 7.42±0.84^a^ |
|  |  | I 2 | 157.57±9.88^b^ | 9.09±0.03^c^ | 0.43±0.05^ab^ | 0.02±0.00^b^ | 1.80±0.26^a^ | 9.55±0.18^c^ | 25.01±0.03^c^ | 9.03±0.68^a^ |
|  |  | I 3 | 83.43±7.48^c^ | 8.32±0.02^e^ | 0.37±0.02^b^ | 0.02±0.00^b^ | 1.85±0.25^a^ | 7.78±0.15^e^ | 24.60±0.02^d^ | 4.67±0.49^b^ |
|  |  | I 4 | 28.20±1.54^d^ | 8.11±0.04^f^ | 0.59±0.04^ab^ | 0.03±0.00^b^ | 3.57±1.46^a^ | 6.71±0.03^f^ | 24.36±0.05^e^ | 0.62±0.04^c^ |
| S-Experiment |  | C0 | 180.85±13.56^c^ | 9.91±0.16^d^ | 0.46±0.06^ab^ | 0.02±0.00^b^ | 3.64±0.77^a^ | 12.55±0.24^cd^ | 17.52±0.44^a^ | 0±0^c^ |
|  |  | V1 | 180.92±6.99^c^ | 10.24±0.02^cd^ | 0.49±0.05^ab^ | 0.02±0.00^a^ | 3.97±0.47^a^ | 12.58±0.10^cd^ | 17.44±0.05^a^ | 0.00±0^d^ |
|  |  | V2 | 197.91±15.39^bc^ | 10.29±0.00^bc^ | 0.46±0.02^ab^ | 0.02±0.00^ab^ | 3.76±0.0.49^a^ | 12.33±0.0.04^d^ | 17.78±0.0.03^a^ | 0.92±0.56^d^ |
|  |  | V3 | 213.92±5.21^ab^ | 10.19±0.20^cd^ | 0.42±0.06^ab^ | 0.02±0.00^ab^ | 3.01±0.81^a^ | 12.34±0.25^d^ | 17.38±0.49^a^ | 2.81±0.47^c^ |
|  |  | P1 | 194.26±11.71^bc^ | 10.59±0.11^ab^ | 0.46±0.04^ab^ | 0.02±0.00^ab^ | 3.82±0.53^a^ | 13.08±0.14^bc^ | 17.63±0.07^a^ | 1.49±0.29^cd^ |
|  |  | P2 | 230.13±4.95^a^ | 10.47±0.02^abc^ | 0.33±0.01^b^ | 0.02±0.00^b^ | 3.33±0.45^a^ | 13.28±0.23^b^ | 17.90±0.01^a^ | 4.77±0.88^b^ |
|  |  | P3 | 216.92±1.63^ab^ | 10.64±0.03^a^ | 0.54±0.08^a^ | 0.02±0.00^ab^ | 3.50±0.81^a^ | 13.88±0.10^a^ | 17.99±0.01^a^ | 7.71±0.42^a^ |

Note: Values represent mean ± SE. I1 (high light), I2, I3 and I4 (low light) in L-Experiment represent 39.5%, 17.1%, 7.1% and 2.8% natural light in the aquaria. 0-3 in S-Experiment represents blank, low, medium and high plant density levels, respectively. V and P represent *V. natans* and *P. maackianus*, respectively. Photosynthetic active radiation (PAR), Total phosphorus (TP), Total nitrogen (TN), Chlorophylla-*a* (Chl-a), Dissolved oxygen (DO).

**Table S3** Crustacean zooplankton functional group and species diversity in seston of S-Experiment.

| Groups | zooplankton density  (ind./L) | zooplankton biomass  (mg/L) | Plelagic (ind./L) | Plant-  associated (ind./L) | Substrate scraper (ind./L) | J | Shannon-  Wiener | Simpson | FRic | FDis | RaoQ | Zp/Chl-*a* |
| --- | --- | --- | --- | --- | --- | --- | --- | --- | --- | --- | --- | --- |
| C0 | 109.50±13.31^ab^ | 0.75±0.07^ab^ | 12.00±3.24^a^ | 0.47±0.12^b^ | 7.05±2.34^a^ | 0.62±0.02^ab^ | 1.15±0.06^a^ | 0.57±0.03^ab^ | 5.29±0.14^a^ | 0.25±0.03^ab^ | 0.11±0.01^ab^ | 1.47±0.06^a^ |
| V1 | 93.70±16.36^ab^ | 0.43±0.06^b^ | 8.56±0.05^a^ | 2.52±0.79^cd^ | 5.60±1.40^a^ | 0.60±0.02^ab^ | 1.00±0.08^a^ | 0.49±0.04^ab^ | 3.90±0.17^b^ | 0.21±0.03^bc^ | 0.08±0.01^b^ | 0.24±0.09^a^ |
| V2 | 141.35±15.69^a^ | 0.71±0.03^ab^ | 10.62±1.23^a^ | 4.18±1.80^ab^ | 1.17±0.38^ab^ | 0.65±0.02^ab^ | 1.12±0.00^a^ | 0.55±0.0.00^ab^ | 4.33±0.0.05^b^ | 0.30±0.01^a^ | 0.13±0.00^a^ | 1.12±0.00^a^ |
| V3 | 98.78±13.62^ab^ | 0.72±0.14^ab^ | 7.62±0.80^a^ | 2.33±0.88^ab^ | 6.71±0.32^a^ | 0.62±0.04^ab^ | 1.15±0.07^a^ | 0.55±0.04^ab^ | 4.52±0.20^b^ | 0.19±0.02^bc^ | 0.07±0.01^b^ | 1.15±0.07^a^ |
| P1 | 108.25±19.26^ab^ | 0.50±0.17^b^ | 7.23±2.75^a^ | 5.58±2.18^a^ | 3.70±1.05^a^ | 0.61±0.02^ab^ | 1.15±0.06^a^ | 0.53±0.03^ab^ | 4.23±0.26^b^ | 0.25±0.01^abc^ | 0.10±0.01^ab^ | 1.15±0.06^a^ |
| P2 | 110.25±16.49^ab^ | 0.52±0.03^ab^ | 13.03±3.54^a^ | 1.32±0.43^ab^ | 6.24±3.01^a^ | 0.72±0.06^b^ | 1.20±0.11^a^ | 0.59±0.06^a^ | 4.19±0.21^b^ | 0.25±0.03^abc^ | 0.10±0.01^ab^ | 1.20±0.11^a^ |
| P3 | 82.84±14.31^b^ | 0.87±0.16^a^ | 1.42±3.39^a^ | 1.84±1.28^ab^ | 8.00±3.51^a^ | 0.53±0.06^b^ | 0.91±0.15^a^ | 0.44±0.07^b^ | 4.33±0.17^b^ | 0.17±4.04^c^ | 0.07±0.02^b^ | 0.91±0.15^a^ |

Note: Values represent mean ± SE. 0-3 represents blank, low, medium and high plant density levels, respectively. V and P represent *V. natans* and *P. maackianus*, respectively. Functional richness (Fric), functional dispersion (FDis), Pielou evenness (J), the ratio of zooplankton biomass and Chl-*a* (Zp/Chl-*a*).

**Table S4** Crustacean zooplankton functional group shaked from the macrophytes of S-Experiment.

| Groups | zooplankton density  (ind./per tank ) | zooplankton biomass  (mg/per tank ) | Plelagic  (ind./per tank) | Plant-associated  (ind./per tank) | Substrate scraper (ind./per tank) |
| --- | --- | --- | --- | --- | --- |
| C0 | 0±0^a^ | 0±0^a^ | 0±0^b^ | 0±0^a^ | 0±0^a^ |
| V1 | 81.00±25.01^a^ | 0.08±0.01^a^ | 0±0^b^ | 0.67±0.674^a^ | 80.33±24.94^a^ |
| V2 | 13.67±4.91^b^ | 0.01±0.00^b^ | 0±0^b^ | 0±0^a^ | 13.67±4.91^b^ |
| V3 | 47.33±16.71^ab^ | 0.03±0.01^b^ | 2. 00±1.00^a^ | 0.33±0.33^a^ | 45.00±15.70^ab^ |
| P1 | 29.67±17.29^b^ | 0.03±0.02^b^ | 1.33±0.88^ab^ | 0±0^a^ | 28.33±16.42^b^ |
| P2 | 5.67±2.67^b^ | 0.01±0.00^b^ | 0±0^b^ | 0.33±0.33^a^ | 5.33±2.85^b^ |
| P3 | 21.67±13.13^b^ | 0.02±0.01^b^ | 0±0^b^ | 0±0^a^ | 21.67±13.13^b^ |

Note: Values represent mean ± SE. 0-3 represents blank, low, medium and high plant density levels, respectively. V and P represent *V. natans* and *P. maackianus*, respectively.

**
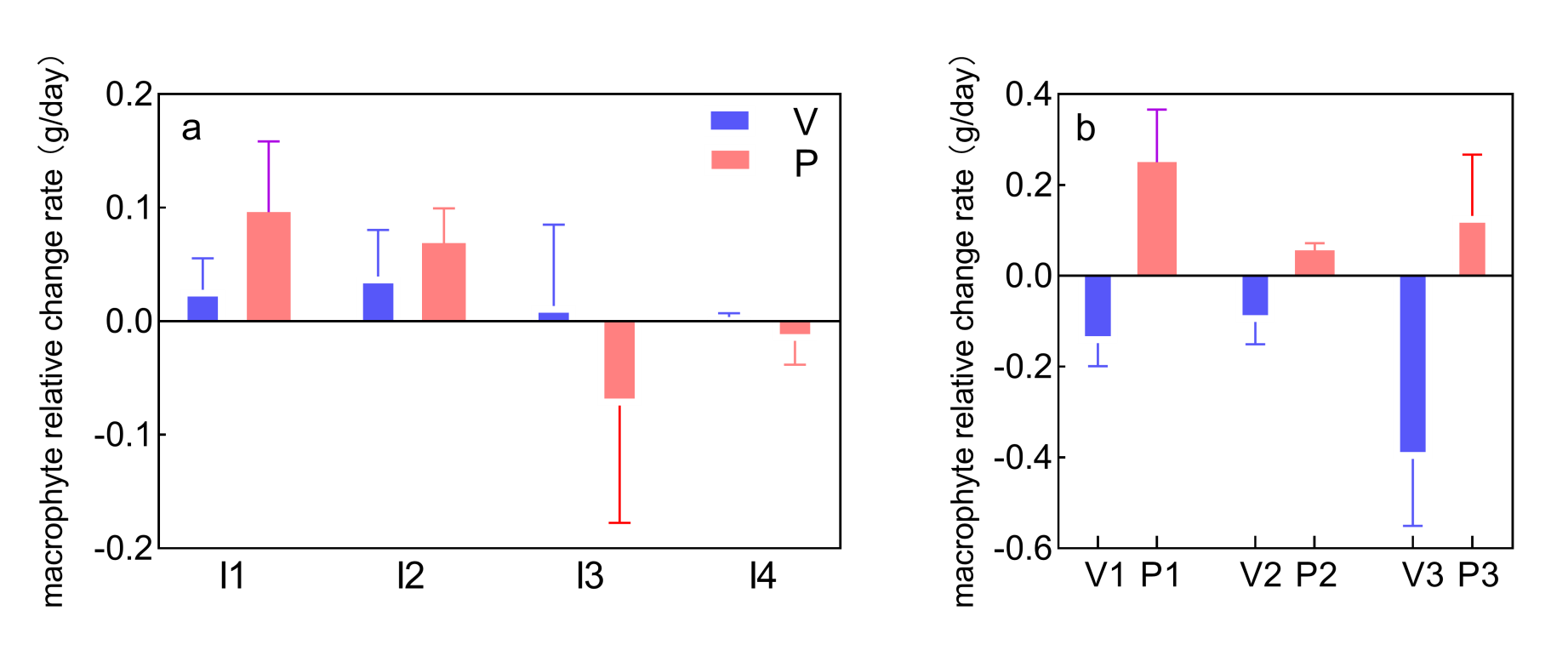
**

**Figure S1** The macrophyte relative change rates in L-Experiment (a) and in S-Experiment (b). Bars represent an average of replicates (±SE). V and P represent *V. natans* and *P. maackianus*, respectively. I1 (high light), I2, I3 and I4 (low light) represent 39.5%, 17.1%, 7.1% and 2.8% natural light in the aquaria. 1-3 represent low, medium and high plant density levels, respectively.

**
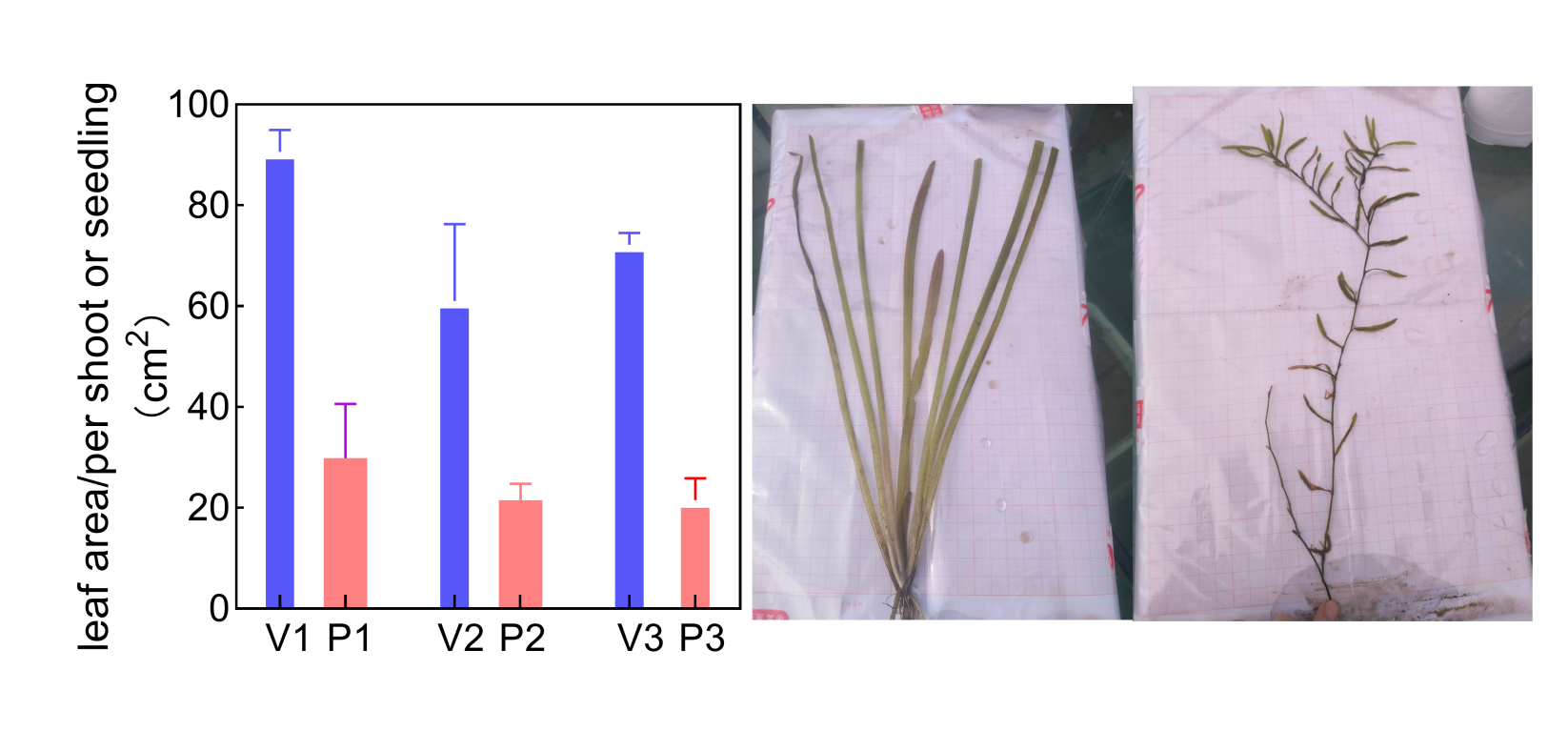
**

**Figure S2** The macrophyte leaf area in S-Experiment. Bars represent an average of replicates ( ±SE). V and P represent *V. natans* and *P. maackianus*, respectively. 1-3 represent low, medium and high plant density levels, respectively.

**
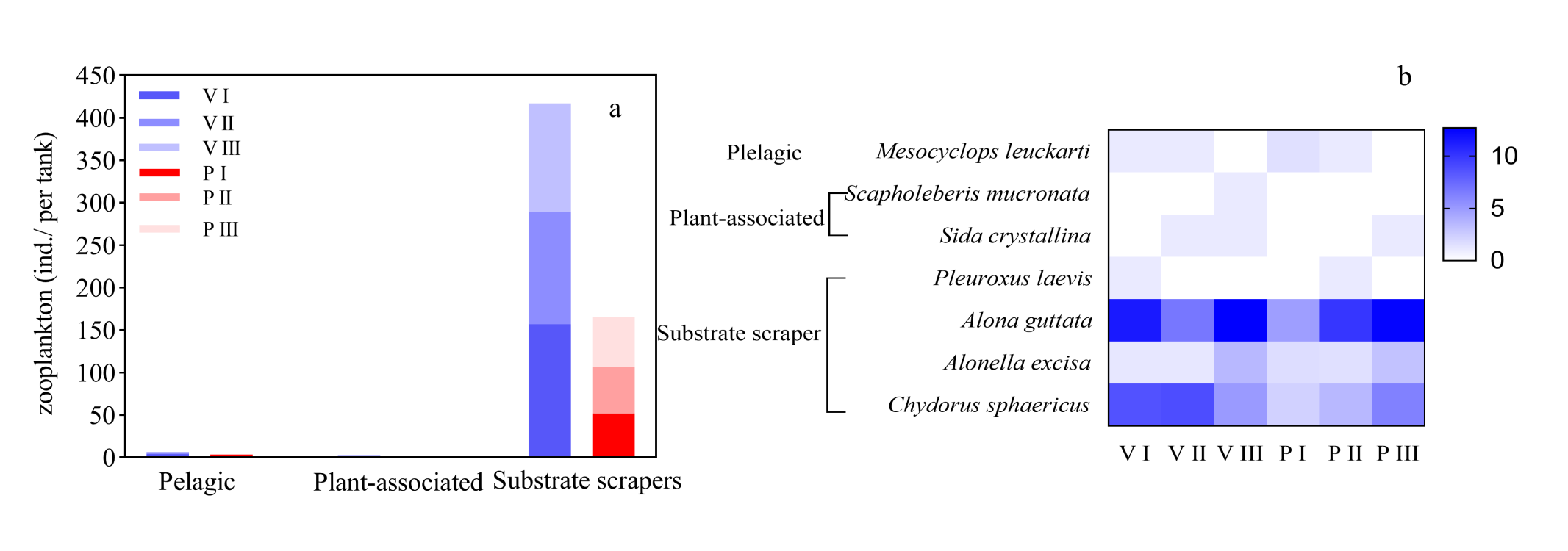
**

**Figure** **S3** The density of zooplankton functional group shaked from the macrophytes in S-Experiment (a). The composition of the shaked zooplankton functional group (b). V and P represent *V. natans* and *P. maackianus*, respectively. I, II and III showed the collected macrophytes were shaken vigorously 30, 60 and 90 times.

**REFERENCES**

Barnett, A. J., Finlay, K., & Beisner, B. E. (2007). Functional diversity of crustacean zooplankton communities: towards a trait‐based classification. Freshwater Biology, 52(5), 796-813.

Braghin, L. D. S. M., Dias, J. D., Simões, N. R., & Bonecker, C. C. (2021). Food availability, depth, and turbidity drive zooplankton functional diversity over time in a Neotropical floodplain. Aquatic Sciences, 83(1), 1-11.

Chen L, Liang J, Qin S, Li L, Yang Y (2016) Determinants of carbon release from the active layer and permafrost deposits on the tibetan plateau. Nat Commun 7: 13046. https://doi.org/10.1038/ncomms13046.

Choi, J. Y., Jeong, K. S., La, G. H., Kim, S. K., & Joo, G. J. (2014). Sustainment of epiphytic microinvertebrate assemblage in relation with different aquatic plant microhabitats in freshwater wetlands (South Korea). J. Limnol, 73(1), 197-202.

Chiang, S.C, Du, N.S., 1979. Fauna Sinica Crustacea: freshwater Cladocera. Beijing: Science Press Academia Sinica (in Chinese).

Rizo, E. Z. C., Gu, Y., Papa, R. D. S., Dumont, H. J., & Han, B. P. (2017). Identifying functional groups and ecological roles of tropical and subtropical freshwater Cladocera in Asia. Hydrobiologia, 799(1), 83-99.

Shen, J., Tai, C., Zhang, Z., Li, D., Song, Chen, G., 1979. Fauna sinica crustacea freshwater copepoda. Institute of Zoology Academia Sinica Beijing: Science Press of China Beijing (in Chinese).
